# Description of Four New *Trypanosoma* Species Infecting Small Wild Mammals from Two Brazilian Biomes: The Pantanal and Cerrado Hotspots

**DOI:** 10.3390/microorganisms13061257

**Published:** 2025-05-29

**Authors:** Arlei Marcili, Andréa Pereira da Costa, Pablo Henrique Nunes, Juliana Isabel Giuli da Silva Ferreira, Renata Tonhosolo, Varley Cardoso Bosco, Isabella Pereira Pesenato, Fábio Fernandes Roxo, Fernanda Aparecida Nieri Bastos, Richard Campos Pacheco, Rogério Vieira Rossi, Thiago Borges Fernandes Semedo, Marina Tiemi Shio, Marcelo Bahia Labruna

**Affiliations:** 1 Departamento de Medicina Veterinária Preventiva e Saúde Animal, Universidade de São Paulo, São Paulo 05508-090, SP, Brazil; ju.gsilva@usp.br (J.I.G.d.S.F.); isa.pesenato@gmail.com (I.P.P.); fenieri@alumni.usp.br (F.A.N.B.); labruna@usp.br (M.B.L.); 2 Programa de Saúde Única, Universidade Santo Amaro, São Paulo 04801-970, SP, Brazil; varleybosco@gmail.com; 3 Programa de Pós-Graduação em Ciência Animal, Universidade Estadual do Maranhão, São Luís 65055-970, MA, Brazil; andrea.costa@professor.uema.br; 4 Instituto Latino-americano da ciências da vida e da Natureza, Universidade Federal da Integração Latino-Americana, Foz do Iguaçu 85867-970, PR, Brazil; pablo.nunes@unila.edu.br; 5 Faculdade de Medicina, Universidade Santo Amaro, São Paulo 97980-000, SP, Brazil; rtonhosolo@prof.unisa.br (R.T.); ffroxo@prof.unisa.br (F.F.R.); 6 Programa de Pos-Graduação em Ciências Veterinárias, Faculdade de Medicina Veterinária, Universidade de Mato Grosso, Cuiabá 78200-000, MT, Brazil; richard@ufmt.br; 7 Laboratório de Mastozoologia, Departamento de Biologia e Zoologia, Instituto de Biociências, Universidade Federal do Mato Grosso, Cuiabá 79560-000, MT, Brazil; rogerrossi@gmail.com; 8 CIBIO, Centro de Investigação em Biodiversidade e Recursos Genéticos, Universidade do Porto, 4150-180 Vairão, Portugal; thiagosemedo@gmail.com; 9 Departamento de Biologia, Universidade do Porto, 4485-653 Vairão, Portugal; 10 BIOPOLIS Program in Genomics, Biodiversity and Land Planning, 4485-653 Vairão, Portugal; 11 Programa em Ciência da Saúde, Universidade Santo Amaro, São Paulo 04801-970, SP, Brazil; mtshio@prof.unisa.br

**Keywords:** taxonomy, *Trypanosoma*, phylogeny, marsupials and rodents

## Abstract

The parasites of the genus *Trypanosoma* have a great diversity of vertebrate hosts and can be transmitted by several groups of invertebrates. All rodent and marsupial species are potential hosts of different trypanosome species. Most species descriptions of trypanosomes have been based only on morphological characteristics. In this study, we conducted a survey on trypanosome infection in small mammals that were caught in an area of the Brazilian Pantanal (Wetlands) and Cerrado (Savanna) biomes in the state of Mato Grosso. The trypanosomes isolated were included in phylogenetic studies based on the SSUrDNA and gGAPDH genes, which were complemented through morphological studies based on scanning and transmission electron microscopy. The morphological and biological discontinuities, along with the phylogenetic position, made it possible to describe four new species of trypanosome hosted by marsupials and rodents, which we described and named as *Trypanosoma daniloi* sp. nov., *Trypanosoma favoritoae* sp. nov., *Trypanosoma percequilloi* sp. nov., and *Trypanosoma trefauti* sp. nov.

## 1. Introduction

The parasites of the genus *Trypanosoma* Gruby, 1843 have a great diversity of vertebrate hosts and can be transmitted by several groups of invertebrates [1,2,3]. According to the way the vector transmits the parasites, trypanosomes are divided into the Stercoraria Section, which is the most reported, and the Salivaria Section, with only two reports [4,5,6].

Concerning trypanosomes’ hosts, the order Rodentia represents approximately 44% of the species within the class Mammalia in Brazil, with 76 genera and 263 species [7]. Metatherians (marsupials), which serve as hosts for trypanosomes, are found in regions such as Australia, New Guinea, Tasmania, and across the Americas [8]. Within the Americas, three distinct orders are recognized: Didelphimorphia, Paucituberculata, and Microbiotheria. Among these, Didelphimorphia stands out as the most diverse and is the only order present in Brazil [9].

All rodent and marsupial species currently described are potential hosts of different trypanosome species [10,11]. Most species descriptions of trypanosomes were based on morphological characteristics and were classified as belonging to the then-called subgenus *Megatrypanum*. Approximately 60 species of trypanosomes have been described in rodents and marsupial species worldwide [1,11,12,13,14,15,16,17,18,19,20,21].

Phylogenetic studies have shown that species of trypanosomes infecting rodents and marsupials are divided into different clades: *T. cruzi* Chagas, 1909; *T. lewisi* (Kent, 1808); Australian trypanosomes; and the Lizard/Snakes/Marsupials Clade [22,23]. DNA sequences obtained directly from the host without parasite isolation have demonstrated that the diversity of rodent and marsupial trypanosomes is still not well known [23,24].

This study involved a survey of trypanosome infections in small mammals captured in regions of the Brazilian Pantanal (Wetlands) and Cerrado (Savanna) biomes, located in the state of Mato Grosso. The isolated trypanosomes were analyzed through phylogenetic studies using the small subunit ribosomal RNA (SSU rDNA) and glyceraldehyde-3-phosphate dehydrogenase (gGAPDH) genes, alongside morphological analyses conducted via scanning and transmission electron microscopy. Based on the observed morphological and biological distinctions, as well as their phylogenetic placement, we identified and described four new trypanosome species infecting marsupials and rodents: *Trypanosoma trefauti* sp. nov., *Trypanosoma favoritoae* sp. nov., *Trypanosoma daniloi* sp. nov., and *Trypanosoma percequilloi* sp. nov.

## 2. Materials and Methods

### 2.1. Trypanosome Isolates

Between 2011 and 2018, 638 wild animals were captured in several Brazilian biomes (Atlantic Forest, Caatinga, Cerrado, Amazon, and Pantanal). Several isolates of parasites of the genus *Trypanosoma* from wild mammals showed distinct growth patterns in axenic culture media and monolayers of cells. The distinct profiles were observed in isolates obtained from rodents and marsupials captured in the state of Mato Grosso from two municipalities: Poconé (16°15′24″ S and 56°37′22″ W) and Chapada dos Guimarães (15°27′38″ S and 55°44′59″ W).

### 2.2. Trypanosoma Isolation and Culture in Mammalian and Insect Cells

Intracardiac blood samples (300 μL) from wild animals were collected in vacutainer tubes containing blood agar (comprising 15% red blood cells from sheep) and in LIT liquid medium supplemented with 20% FBS [24]. The isolates were cryopreserved in liquid nitrogen in the collection “Coleção Brasileira de Tripanossomatídeos” (CBT), in the Department of Preventive Veterinary Medicine and Animal Health, School of Veterinary Medicine and Animal Science, University of São Paulo, Brazil. Additionally, blood samples were fixed in ethanol (primary sample) for molecular detection and smeared on glass slides for morphological studies. The cryopreserved cultures were thawed to assess the viability of the cryopreservation process.

Parasites were used to infect mammalian kidney epithelial cells (VERO) and insect cells (C6/36 and SF9). Cells were kept in culture comprising RPMI (Invitrogen, Waltham, MA, USA) medium supplemented with 10% FBS, 10,000 units/mL of penicillin (Sigma, St. Louis, MI, USA), and 10,000 µg/mL of streptomycin (Sigma) at 37 °C, and insect cells (SF9) were cultivated using TC100 supplemented with 10% FBS (Invitrogen) in a greenhouse at 25–28 °C. These cultures were used to perform scanning and transmission electron microscopy [25]. Axenic media (LIT, RPMI, and TC100) supplemented with 10% SFB were used for parasite growth.

Growth in the culture media was considered to have occurred when an increase in the number of epimastigote and culture-derived trypomastigote forms was observed, along with sustained growth after five passages in the same culture medium or cell monolayer. Parasites were considered not to have grown when there was a decrease in epimastigote forms, leading to their complete disappearance.

### 2.3. Morphological Characterization

#### 2.3.1. Scanning Electron Microscopy

Parasites grown in insect and mammalian cells were fixed with Karnovsky (*v*/*v*) in 0.1 M cacodylate buffer (pH 7.4) for 2 h. Glass slides treated with 0.1% poly-L-lysine were covered with fixed cells, followed by dehydration in a graded series of acetone. Dehydration was completed in the Critical Point Dryer Balzers CPD 030. The coverslips containing the material were covered with gold in a sputter coater (Balzers SCD 050). Samples were scanned using a HITACHITM3000 SEM device (HITACHI, Tokyo, Japan).

#### 2.3.2. Transmission Electron Microscopy

The material fixed in 2.5% glutaraldehyde in phosphate buffer was post-fixed with 1% *v*/*v* osmium tetroxide in 50 mM cacodylate buffer (pH 7.4) and stained with uranyl acetate for 12 h [25]. Subsequently, the samples were dehydrated in acetone and embedded in Araldite-EPON resin for 12 h at 60 °C. Grids were stained with uranyl acetate and lead citrate for 45 min and 10 min, respectively. The grids were then observed on a Philips CM 100 TEM at the Biology Department of the Institute of Biosciences, UNESP, Rio Claro campus, Rio Claro, São Paulo, Brazil.

### 2.4. Molecular and Phylogenetic Analysis

DNA was extracted and purified from the trypanosome cultures and the primary samples (blood preserved in ethanol) using the DNA Purelink Genomic DNA Kit (Thermo Fisher Scientific, Waltham, MA, USA) and quantified using a Nanodrop spectrophotometer (Thermofisher).

The DNA samples were subjected to the conventional polymerase chain reaction (PCR) for classical genes for phylogenetic inferences in the Tripanosomatidae family, the full-length SSU rDNA (~2200 bp) and gGAPDH genes (~900 bp) following [26,27,28]. The thermo-cycler profile consisted of an initial denaturation (4 min at 95 °C) followed by 30 cycles of chain denaturation (30 s at 95 °C), primer hybridization (30–60 s at 52–54 °C), and nucleotide extension (30–60 s at 72 °C). PCR products of the expected size (which size?) were visually identified on a 1% agarose gel and then purified using ExoSap-IT! (USB Corporation, Cleveland, OH, USA) following the manufacturer’s instructions. The purified PCR products were sequenced in an automatic sequencer (Applied Biosystems/Perkin-Elmer ABI PRISM 310 Genetic Analyzer, Foster City, CA, USA) in accordance with the manufacturer’s recommendations.

The consensus sequences for each individual gene were assembled from chromatograms of the forward and reverse sequences using Geneious software v7.1.7 (Biomatters Ltd., Auckland, New Zealand). The obtained sequences in this study have been deposited in GenBank (accession numbers in Appendix A).

The sequences obtained for each gene were aligned separately with sequences previously determined for other trypanosomatid species available in GenBank (Appendix A), using Clustal X [10], and were adjusted manually using GeneDoc [29]. The alignments were rigorously inspected for any obvious misalignments; however, no corrections were necessary. To detect potential cases of sequencing errors due to contamination or paralogy, the alignment for each gene was analyzed by maximum likelihood (ML) and rapid bootstrapping using RAxML v8.019, comparing the monophyletic results with traditional taxonomy; however, no paralogy was detected in our data [30]. To evaluate the occurrence of substitution saturation, we used the index of substitution saturation (Iss) test as described by Xia et al. (2003) and Xia and Lemey (2009), implemented in the software Dambe 5.3.38 [31,32,33]. Alignments of both loci (i.e., SSU rDNA and gGAPDH) were concatenated into a single 2874 base pairs matrix in which phylogenetic analyses were performed. We did not use partitioning in the phylogenetic analysis of our dataset.

The phylogenetic hypotheses were inferred using two reconstruction methods. First, maximum likelihood (ML) phylogenetic reconstructions were performed using RAxML to estimate the best tree via the command line with the “a” algorithm for rapid bootstrapping analysis (ML search and bootstrapping) in a single step, specifying a random seed for the parsimony estimation [30]. The number of alternative runs was set to 10, and the analyses were performed under the GTR model with a Gamma distribution. The concatenated alignment of both genes was also used to perform bootstrap replicates using the autoMRE function for the extended majority-rule consensus tree criterion available in RAxML v8 [34] to assess support for individual nodes. This option allowed the bootstrap convergence test to be conducted, which determines whether the number of replicates is sufficient to obtain stable support values [35].

Bayesian analysis of the unpartitioned concatenated alignment was performed using ExaBayes V1.4 [36]. We conducted two independent runs, each with two chains (one cold and one heated) for 1,000,000 generations using the GTR + G model. The tree space was sampled every 100 generations to yield a total of 10,001 trees. Parameter estimates and ESS values were visualized in Tracer v 1.6, and the last 7500 trees were sampled after confirming convergence [37]. This allowed us to visualize the log of the posterior probabilities within and between independent runs and to ensure that the average standard deviation of split frequencies was <1%, effective sample sizes (ESSs) were >200, and the potential scale reduction factor for estimated parameters was approximately 1.0. We generated the 50% most credible set of trees from the posterior distribution of possible topologies using the consensus algorithm of ExaBayes (burn-in: 25%; thinning: 500).

## 3. Results

A total of ten isolates were obtained from rodents (*Hylaeamys megacephalus*) and marsupials (*Philander canus* and *Didelphis albiventris*). The infected animals were sampled in Mato Grosso state in two municipalities: Poconé (Pantanal biome) and Chapada dos Guimarães (Cerrado biome) (Table 1). The species description was based solely on already established cultures.

During the isolation of *Trypanosoma* parasites, the cultures presented different patterns of nutritional requirements. The isolates CBT127, CBT228, CBT229, CBT230, CBT231, CBT232, CBT233, and CBT272 grew in the VERO cell monolayer with RPMI medium. However, CBT51 grew only in the SF9 cell monolayer with TC100 medium (Table 2) and exhibited partial growth in the axenic TC100 medium. The isolate CBT52 was the only one able to grow in the axenic LIT medium. None of the isolates grew in the axenic RPMI medium.

Morphological analyses were performed only with epimastigotes or trypomastigotes present in culture. The blood trypomastigote form was not detected in blood smears due to the low parasitemia present in the hosts.

**Figure 1:**
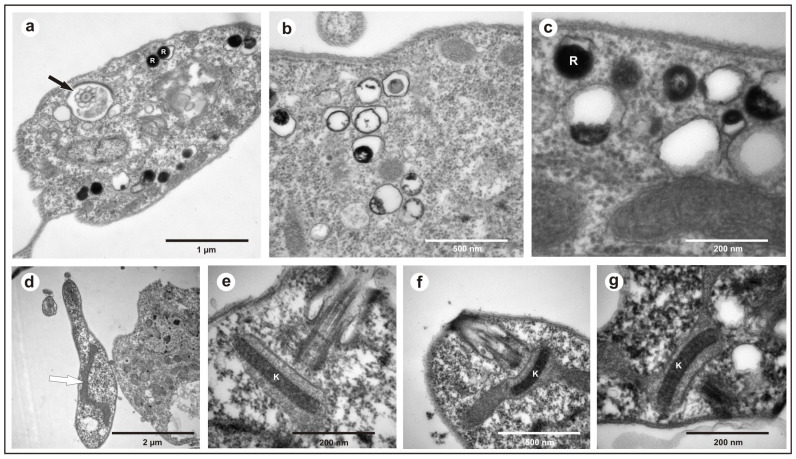
Transmission electron microscopy of *Trypanosoma trefauti* obtained in the state of Mato Grosso: (**a**) flagellum; (**b**,**c**) reservosomes; (**d**) mitochondria; (**e**–**g**) kinetoplast. The black arrow indicates the flagellum, and the white arrow indicates the mitochondria. K—kinetoplast; R—reservosomes.

All the obtained isolates exhibited a free flagellum (Figure 1a), the presence of a single mitochondrion (Figure 1d), a well-compacted kinetoplast (Figure 1e–g), and the presence of reservosomes (Figure 1b,c), morphological characteristics common to various species of the genus *Trypanosoma*.

**Figure 2:**
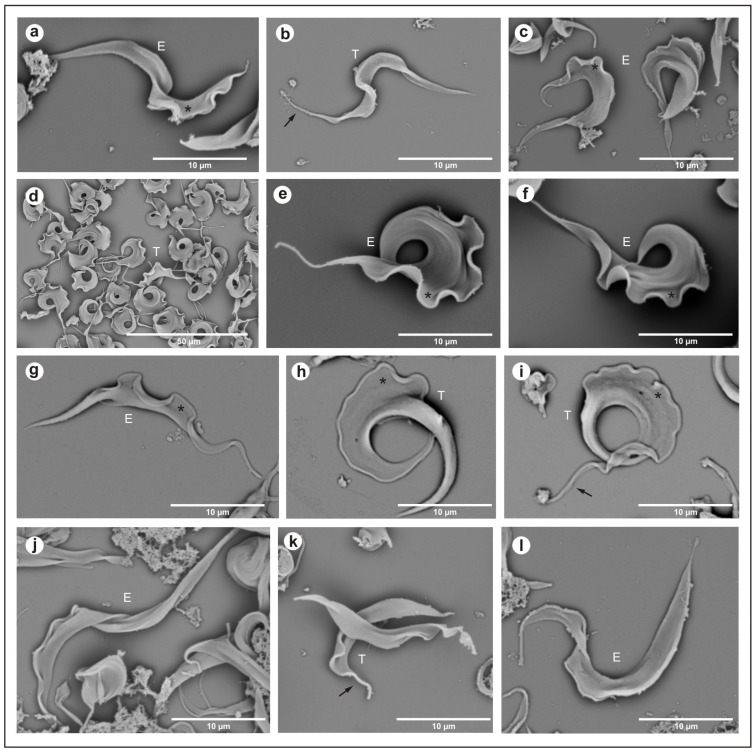
Scanning electron microscopy: (**a**–**c**) *Trypanosoma trefauti* (CBT 51); (**d**–**f**) *Trypanosoma favoritoae* (CBT 52); (**g**–**i**) *Trypanosoma daniloi* (CBT127); (**j**–**l**) *Trypanosoma percequilloi* (CBT 228). The data show epimastigote (E) and trypomastigote (T) forms of the parasites. The black arrow indicates the flagellum, and the asterisk (*) denotes the undulating membrane. E—epimastigote; T—trypomastigote.

In the isolate from *Hyalaemys megacephalus*, *Trypanosoma trefauti* sp. nov., during the stationary phase, epimastigotes were large and wide and had little motility, with a free flagellum (Figure 2a–d) and a large undulating membrane. Trypomastigotes were shorter and slenderer than epimastigote forms, with a poorly developed undulating membrane and a short free flagellum (Figure 2a–c).

In the isolate from *Philander canus*, *Trypanosoma favoritoae* sp. nov., during the stationary phase, the epimastigotes were very large, with a well-developed undulating membrane and a motile free flagellum (Figure 2g–i). Trypomastigotes forms were not observed in blood smears or stationary cultures.

In the isolate from *Didelphis albiventris*, *Trypanosoma daniloi* sp. nov., during the stationary phase, epimastigotes were large and slender and had little motility, with a free flagellum (Figure 2a–d) and a large undulating membrane. Trypomastigotes were large and wide, with a very well-developed undulating membrane and short free flagellum, and the posterior portion was well developed (Figure 2j–l).

The isolates from *Hyalaemys megacephalus*, *Trypanosoma percequilloi* sp. nov., showed similar morphology and development in culture compared to *T. daniloi*. During the stationary phase, epimastigotes were very large and wide and had short motility with a free flagellum (Figure 2a–d) and an undulating membrane. Trypomastigotes were shorter than epimastigote forms, with short motility and a free flagellum, and a short undulating membrane (Figure 2d–f).

### 3.1. Taxonomy of Trypanosoma

Phylum: Euglenozoa Cavalier-Smith, 1981

Class: Kinetoplastea Konigberg 1963

Order: Trypanosomatida (Kent 1880) Holande, 1952

Family: Trypanosomatidae Doflein, 1901

*Trypanosoma daniloi* sp. nov. Marcili

*Trypanosoma favoritoae* sp. nov. Marcili

*Trypanosoma percequilloi* sp. nov. Marcili

*Trypanosoma trefauti* sp. nov. Marcili

 

***Trypanosoma daniloi* sp. nov. Marcili**

*Morphology (n = one isolate)*

The epimastigotes were large and slender and had little motility, with a free flagellum and a large undulating membrane. Trypomastigotes were large and wide, with a very well-developed undulating membrane, a short free flagellum, and a well-developed posterior portion. No trypomastigote forms were observed in the host blood smear.

 

*Host type*

Mammalia, Didelphimorphia, Didelphidae, *Didelphis albiventris* (Lund 1840).

 

*Vector*

Unknown.

 

*Type locality*

The specimen was captured in the Cerrado biome, in the municipality of Chapada dos Guimarães (15°27′38″ S and 55°44′59″ W) in Mato Grosso state, Brazil.

 

*Type material*

Hapantotype: CBT isolate 127. The cultures were deposited in the Brazilian Collection of Trypanosomatids (CBT).

 

*Gene sequences*

The *Trypanosoma daniloi* hapantotype CBT 127’s SSUrDNA sequence was deposited in GenBank under the accession number MZ352973, and the gGAPDH sequence was deposited under the accession number MZ351927.

 

*Etymology*

The name *Trypanosoma daniloi* was given in honor of researcher and friend Danilo Gonçalves Saraiva (in memoriam), who was always passionate about wildlife.

 

 

***Trypanosoma favoritoae* sp. nov. Marcili**

*Morphology (n = one isolate)*

The epimastigotes were very large, with a well-developed undulating membrane and a motile free flagellum. Trypomastigotes forms were not observed in blood smears or stationary cultures.

 

*Host type*

Mammalia, Didelphimorphia, Didelphidade, *Philander canus* (Linnaeus, 1758).

 

*Vector*

Unknown.

 

*Type locality*

The specimen was captured in the Pantanal biome, in the municipality of Poconé (16°15′24″ S and 56°37′22″ W) in Mato Grosso state, Brazil.

 

*Type material*

Hapantotype: CBT isolate 52. The cultures were deposited in the Brazilian Collection of Trypanosomatids (CBT).

 

*Gene sequences*

The *Trypanosoma favoritoae* hapantotype CBT 52’s SSUrDNA sequence was deposited in GenBank under the accession number MZ352972, and the gGAPDH sequence was deposited under the accession number MZ351926.

 

*Etymology*

The name *Trypanosoma favoritoae* was given in honor of Dr. Sandra Elisa Favorito-Raimo, PhD, an ichthyologist who inspired scientific interest in a generation of undergraduate students.

 

***Trypanosoma percequilloi* sp. nov. Marcili**

*Morphology (n = seven isolates)*

The epimastigotes were very large and wide and had short motility with a flagellum and an undulating membrane. Trypomastigotes were shorter than epimastigote forms, large, with short motility, a flagellum, and an undeveloped undulating membrane. No trypomastigote forms were observed in the host blood smear.

 

*Host type*

Mammalia, Rodentia, Sigmodontinae, *Hylaeamys megacephalus* (Fischer, 1814).

 

*Vector*

Unknown.

 

*Type locality*

The specimen was captured in the Pantanal biome, in the municipality of Poconé (16°15′24″ S and 56°37′22″ W) in Mato Grosso state, Brazil.

 

*Type material*

Hapantotype: isolate CBT 228. Paratypes: isolates CBT 229, CBT 230, CBT 231, CBT 232, CBT 233, and CBT 272. The cultures were deposited in the Brazilian Collection of Trypanosomatids (CBT).

 

*Gene sequences*

The *Trypanosoma percequilloi* hapantotype CBT 228’s SSUrDNA sequence was deposited in GenBank under the accession number MZ352974, and the gGAPDH sequence was deposited under the accession number MZ351928. For the paratypes CBT 229, CBT 230, CBT 231, CBT 232, CBT 233, and CBT 272, respectively, the SSUrDNA sequences were deposited in GenBank as MZ352975, MZ352976, MZ352977, MZ352978, MZ352979, and MZ352980, and the gGAPDH sequences were deposited as MZ351929, MZ351930, MZ351931, MZ351932, MZ351933, and MZ351934.

 

*Etymology*

The name *Trypanosoma percequilloi* was given in honor of Dr. Alexandre Reis Percequillo, PhD, a mammalogist who contributed to the description and review of Brazilian rodent diversity.

 

***Trypanosoma trefauti* sp. nov. Marcili**

*Morphology (n = one isolate)*

Epimastigotes were large and broad and had low motility, with a free flagellum and a large undulating membrane. Trypomastigotes were shorter and slenderer than the epimastigote forms, with a poorly developed undulating membrane and a short free flagellum. No trypomastigote forms were observed in the host blood smear.

 

*Host type*

Mammalia, Rodentia, Sigmodontinae, *Hylaeamys megacephalus* (Fischer, 1814).

 

*Vector*

Unknown.

 

*Type locality*

The specimen was captured in the Pantanal biome, in the municipality of Poconé (16°15’24″ S and 56°37’22″ W) in Mato Grosso state, Brazil.

 

*Type material*

Hapantotype: CBT isolate 51. The cultures were deposited in the Brazilian Collection of Trypanosomatids (CBT).

 

*Gene sequences*

The *Trypanosoma trefauti* hapantotype CBT 51 SSUrDNA sequence was deposited in GenBank under the accession number MZ352971, and the gGAPDH sequence was deposited under the accession number MZ351925.

 

*Etymology*

The name *Trypanosoma trefauti* was given in honor of Dr. Miguel Rodrigues Trefaut, PhD, a herpetologist responsible for describing and reviewing Brazil’s biodiversity.

### 3.2. Phylogenetic Analysis

**Figure 3:**
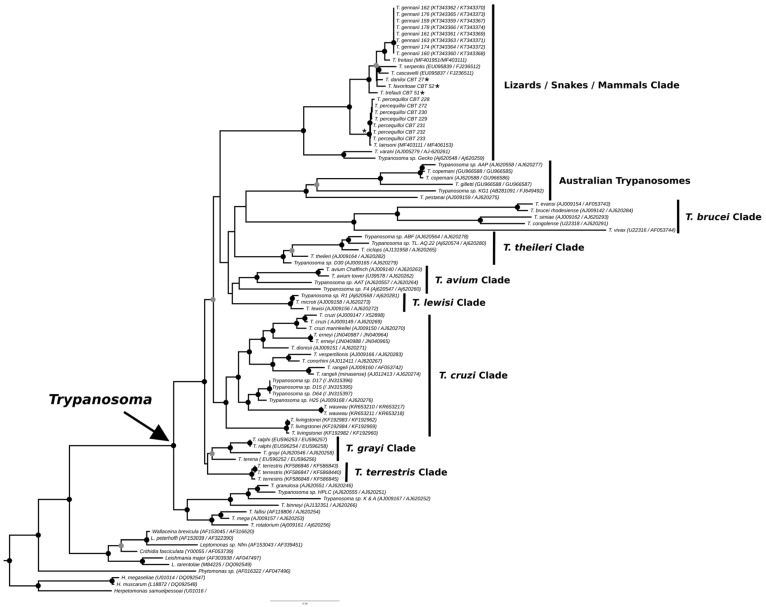
Best tree of maximum likelihood analysis of the *Trypanosoma* genus obtained based on the SSUrDNA gene concatenated with the gGAPDH gene, with *Herpetomonas* as outgroup (2874 characters, 973 informative sites). Black dots indicate node support values higher than >75%, and gray dots indicate support values higher than 50% from 1000 bootstrap pseudoreplicates. Those with asterisks denote the four new species described in this study.

**Figure 4:**
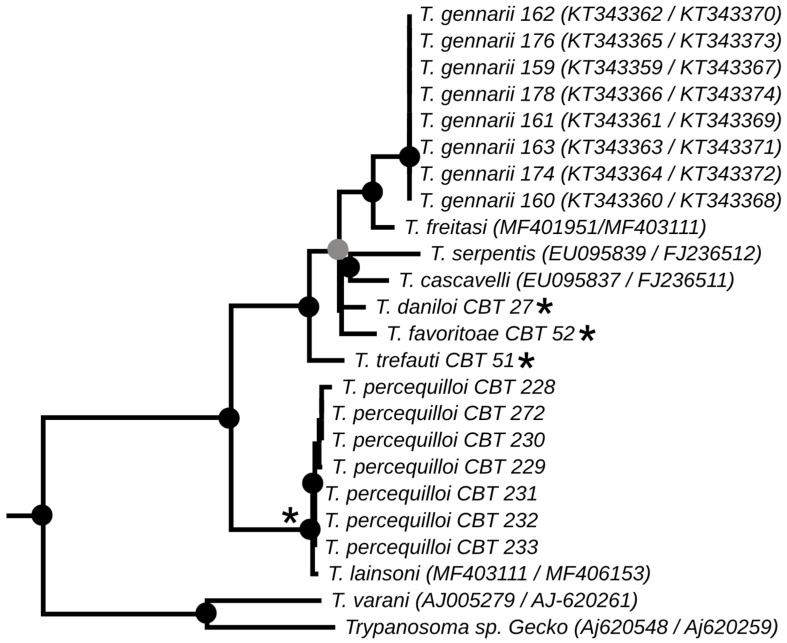
Best tree of maximum likelihood analysis showing the Lizard/Snake/Mammals clade obtained based on the SSUrDNA gene concatenated with the gGAPDH gene. Black dots indicate node support values higher than >75%, and gray dots indicate support values higher than 50% from 1000 bootstrap pseudoreplicates. Those with asterisks denote the four new species described in this study.

**Figure A1:**
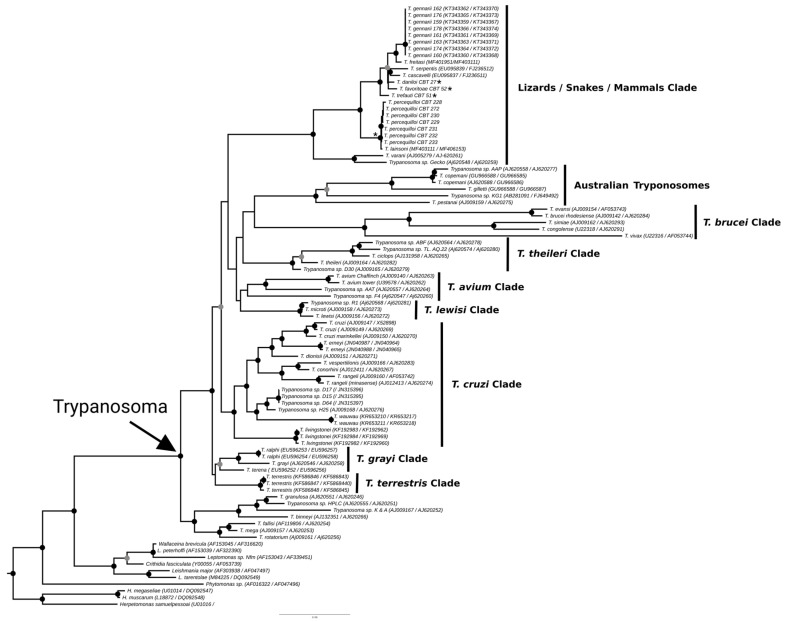
Bayesian phylogenetic reconstruction of the concatenated alignment of SSU rDNA and gGAPDH genes performed using ExaBayes of the *Trypanosoma* genus. Black dots indicate node support values higher than >75%, and gray dots indicate support values higher than 50% from 1000 bootstrap pseudoreplicates. Those with asterisks denote the four new species described in this study.

The phylogenies obtained from the concatenated analysis of the SSU rDNA and gGAPDH genes using the maximum likelihood (Figure 3) and Bayesian methods (Appendix A Figure A1) revealed identical topologies in both methods, except for the positioning of the species *T. favoritae* and *T. daniloi*, which formed a sister group in ML analysis, but in Bayesian analysis, *T. daniloi* was positioned as a sister group to *T. freitasi* Rego, Magalhães e Siqueira, 1957 plus *T. gennarii* Marcili, 2017. (Figure 4). Despite this divergence, our phylogenetic results corroborate the phylogenetic relationships already described for the *Trypanosoma* genus, with well-supported clades (100% bootstrap and 1.0 posterior probability) [38]. All new isolates were grouped into the Lizard/Snakes/Mammals Clade (Figure 4). Three subclades were formed: one composed of the species that parasitize lizards (*T. varani* Wenyon, 1908 and *Trypanosoma* sp. Gecko) (100% bootstrap and 1.0 posteriori probability); another composed of *T. lainsoni* Naiff and Barrett, 2013 and *T. percequilloi* sp. nov. (100% of bootstrap and 1.0 posteriori probability); and a group consisting of *T. trefauti* sp. nov., *T. favoritoae* sp. nov., *T. daniloi* sp. nov., *T. freitasi*, *T. gennarii*, and snake trypanosomes (*T. cascavelli* and *T. serpentis*) (100% bootstrap and 1.0 posteriori probability). The new trypanosome species showed sequence divergence values ranging from 1.4% to 4.3% and high branch support values (100% bootstrap and 1.0 posteriori probability).

## 4. Discussion

The identification, characterization, and evaluation of parasites are essential for strategic actions in biodiversity conservation and human health prevention. In the present study, we described four new species of trypanosomes found in marsupials and rodents, based on morphological, molecular, and phylogenetic analyses. These species, named *Trypanosoma trefauti* sp. nov., *Trypanosoma favoritoae* sp. nov., *Trypanosoma daniloi* sp. nov., and *Trypanosoma percequilloi* sp. nov., are all positioned within the Lizard/Snakes/Mammals Clade (Figure 4).

Numerous trypanosome species have been identified in wildlife across different parts of the world, including Brazil [21,23,24,27,39,40]. Phylogenetic analyses using SSU rDNA and gGAPDH gene sequences have proven effective for determining the evolutionary relationships among species, genera, and within the Trypanosomatidae family. These molecular tools have enabled the classification of species into distinct clades, often reflecting their association with specific vertebrate hosts [1,3,12,15,28,41,42,43,44,45].

An important point is that we still know nothing about the biology of these new parasites in nature, nor about their vectors. The findings of sandflies infected by a trypanosome from lizards (*T. varani*) and a trypanosome phylogenetically related to those identified in snakes suggest that sandflies are possible vectors of trypanosomes from this clade [26,46]. In Brazil, sandflies have been reported in association with possum nests, and rodents and marsupials have been identified as food sources for sandflies. These findings indicate that new trypanosome species can be transmitted by sandflies [47,48].

In Didelphimorphia, two new species were isolated from *Phylander opossum* (*Trypanosoma favoritoae* sp. nov.) and *Didelphis albiventris* (*Trypanosoma daniloi* sp. nov.). Because these marsupials use all forest strata, are nomadic, and omnivorous, they are exposed to several trypanosomes in the natural environment, including all transmission cycles of *T. cruzi* [49,50]. In fact, these marsupials are known reservoirs of numerous other species of *Trypanosoma,* such as *T. rangeli*, *T. freitasi*, *T. gennarii*, *T. janseni*, *T. dionisii*, and *T. cascavelli* [22,51,52,53,54]. Of these species, *T. freitasi*, *T. gennarii*, and *T. janseni* have been described only in Didelphimorphia.

Studies related to the role of rodents as reservoirs of trypanosomatids have documented different species of *Trypanosoma,* which are fundamental for the analysis of the transmission dynamics of these protozoa in nature. For instance, previous studies have reported infection in rodents by *T. rochasilvai*, *T. cruzi*, *T. amileari, T. evansi*, *T. lewisi*, *T. musculi*, and *Trypanosoma lainsoni* [51,55,56,57,58,59,60,61].

The continuous process of environmental degradation in Pantanal and Cerrado biomes, such as continuous burning and habitat loss, combined with the absence of environmental protection policies, puts endemic biodiversity at risk in these biomes, as well as the parasite fauna associated with these species [62,63]. Consequently, the loss of biodiversity leads to changes in the ecology of the remaining species, including their parasites; the restriction of habitat and food; and greater contact between wild animals and domestic animals and humans, increasing pathogen transmission and the incidence of diseases [64,65].

Additionally, small rodents and marsupials are considered good environmental bioindicators because they have large populations, short life cycles, and are found in all biomes and habitats, both natural and modified, responding quickly to environmental changes [66]. Moreover, the biomes (Pantanal and Cerrado) where the hosts were captured are undergoing significant anthropogenic impacts, with substantial loss of territory. Habitat loss affects the diversity of hosts and their associated microorganisms. They are also important prey for most small and medium-sized carnivores, which enhances the transmission of pathogens. However, further investigations, especially regarding the ecology and habitat of small mammals, are necessary to clarify the potential risks of infection by these trypanosomes in humans and domestic animals.

## Figures and Tables

**Table 1:** Isolated *Trypanosoma*, genetic code, host, and geographic location of the infected wild animals.

*Trypanosoma* spp.	Isolate Code	Host	Geographic Source	
*Trypanosoma trefauti*	CBT 51	*Hylaeamys megacephalus*	Poconé	MT
*Trypanosoma favoritoae*	CBT 52	*Phylander canus*	Poconé	MT
*Trypanosoma daniloi*	CBT 127	*Didelphis albiventris*	Chapada Guimarâes	MT
*Trypanosoma percequilloi*	CBT 228	*Hylaeamys megacephalus*	Poconé	MT
*Trypanosoma percequilloi*	CBT 229	*Hylaeamys megacephalus*	Poconé	MT
*Trypanosoma percequilloi*	CBT 230	*Hylaeamys megacephalus*	Poconé	MT
*Trypanosoma percequilloi*	CBT 231	*Hylaeamys megacephalus*	Poconé	MT
*Trypanosoma percequilloi*	CBT 232	*Hylaeamys megacephalus*	Poconé	MT
*Trypanosoma percequilloi*	CBT 233	*Hylaeamys megacephalus*	Poconé	MT
*Trypanosoma percequilloi*	CBT 272	*Hylaeamys megacephalus*	Poconé	MT

Data showing designation of four new *Trypanosome* species and their respective genetic codes, as well as the species of the infected animals and their geographic location. MT—Mato Grosso; CBT—Brazilian Collection of Trypanosomatids.

**Table 2:** Growth conditions of *Trypanosoma* isolates from rodents and marsupials.

Isolates	Media or Cells Monolayer Culture				
(CBT)	LIT	TC100	RPMI	Vero + RPMI	SF9 + TC100
51	-	+/-	-	-	+
52	+	-	-	-	-
127	-	-	-	+	-
228	-	-	-	+	-
229	-	-	-	+	-
230	-	-	-	+	-
231	-	-	-	+	-
232	-	-	-	+	-

Blood cultures were applied to the field isolation medium (LIT). The medium was used to infect mammalian cell monolayers (VERO) grown in RPMI medium supplemented with 10% FBS at 37 °C and insect cell monolayers (SF9) grown in TC100 supplemented with 10% FBS at 25–28 °C. Parasites were tested in culture media and cell monolayers. “+” denotes growth, and “-” denotes no growth under the tested conditions.

## Data Availability

The original contributions presented in this study are included in the article. Further inquiries can be directed to the corresponding author.

## References

[1] Hamilton P.B., Stevens J.R., Gaunt M.W., Gidley J., Gibson W.C. (2004). Trypanosomes are monophyletic: Evidence from genes for glyceraldehyde phosphate dehydrogenase and small subunit ribosomal RNA. Int. J. Parasitol..

[2] Simpson A.G., Stevens J.R., Lukeš J. (2006). The evolution and diversity of kinetoplastid flagellates. Trends Parasitol..

[3] Hamilton P.B., Gibson W.C., Stevens J.R. (2007). Patterns of co-evolution between trypanosomes and their hosts deduced from ribosomal RNA and protein-coding gene phylogenies. Mol. Phylogenetics Evol..

[4] Hoare C.A. (1972). The Trypanosomes of Mammals.

[5] Rodrigues A.C., Garcia H.A., Batista J.S., Minervino A.H.H., Góes-Cavalcante G., DA Silva F.M., Ferreira R.C., Campaner M., Paiva F., Teixeira M.M.G. (2010). Characterization of spliced leader genes of *Trypanosoma* (*Megatrypanum*) *theileri*: Phylogeographical analysis of Brazilian isolates from cattle supports spatial clustering of genotypes and parity with ribosomal markers. Parasitology.

[6] Milocco C., Kamyingkird K., Desquesnes M., Jittapalapong S., Herbreteau V., Chaval Y., Douangboupha B., Morand S. (2013). Molecular demonstration of *Trypanosoma evansi* and *Trypanosoma lewisi* DNA in wild rodents from Cambodia, Lao PDR and Thailand. Transbound. Emerg. Dis..

[7] Srbek-Araujo A.C. (2018). Do female jaguars (*Panthera onca* Linnaeus, 1758) deliberately avoid camera traps?. Mamm. Biol..

[8] Musser G.C., Carleton M.D., Wilson D.E., Reeder D.M. (2005). Superfamily Muroidea. Mammal Species of the World: A Taxonomic and Geographic Reference.

[9] Wilson D.E., Reeder D.M. (2005). Mammal Species of the World: A Taxonomic and Geographic Reference.

[10] Thompson J.D., Gibson T.J., Plewniak F., Jeanmougin F., Higgins D.G. (1997). The CLUSTAL_X windows interface: Flexible strategies for multiple sequence alignment aided by quality analysis tools. Nucleic Acids Res..

[11] DA Silva F.M., Rodrigues A.C., Campaner M., Takata C.S.A., Brigido M.C., Junqueira A.C.V., Coura J.R., Takeda G.F., Shaw J.J., Teixeira M.M.G. (2004). Randomly amplified polymorphic DNA analysis of *Trypanosoma rangeli* and allied species from humans, monkeys, and other sylvatic mammals of the Brazilian Amazon disclosed a new group and a species-specific marker. Parasitology.

[12] Rêgo S.F.M., Magalhães A.E.A., Siqueira A.F. (1957). Um novo tripanossoma do gambá, *Trypanosoma freitasi* sp. nov. Rev. Bras. Malariologia.

[13] Deane M.P., Milder R. (1972). Ultrastructure of the “cyst-like bodies” of *Trypanosoma conorhini*. J. Protozool..

[14] Mello D.A. (1977). *Trypanosoma (Megatrypanum)* samueli sp. nov., a Trypanosomatidae isolated from *Monodelphis domesticus* (Wagner, 1842) (Marsupiala). Ann. Parasitol. Hum. Comparée.

[15] Noyes H., Stevens J., Teixeira M., Phelan J., Holz P. (1999). A nested PCR for the ssrRNA gene detects *Trypanosoma binneyi* in the platypus and *Trypanosoma* sp. in wombats and kangaroos in Australia. Int. J. Parasitol..

[16] Gurgel-Gonçalves R., Ramalho E.D., Duarte M.A., Palma A.R.T., Abad-Franch F., Carranza J.C., Cuba C.A.C. (2004). Enzootic transmission of *Trypanosoma cruzi* and *T. rangeli* in the Federal District of Brazil. Rev. Inst. Med. Trop. Sao Paulo.

[17] Lainson R., Da Silva F., Franco C. (2008). *Trypanosoma (Megatrypanum)* saloboense sp. nov. (Kinetoplastida: Trypanosomatidae) parasite of *Monodelphis emiliae* (Marsupiala: Didelphidae) from Amazonian Brazil. Parasite.

[18] McINNES L.M., Gillett A., Ryan U.M., Austen J., Campbell R.S.F., Hanger J., Reid S.A. (2009). *Trypanosoma irwini* n. sp (Sarcomastigophora: Trypanosomatidae) from the koala (*Phascolarctos cinereus*). Parasitology.

[19] Austen J.M., Jefferies R., Friend J.A., Ryan U., Adams P., Reid S.A. (2009). Morphological and molecular characterization of *Trypanosoma copemani* sp. nov. (Trypanosomatidae) isolated from Gilbert’s potoroo (*Potorous gilbertii*) and quokka (*Setonix brachyurus*). Parasitology.

[20] McInnes L.M., Hanger J., Simmons G., Reid S.A., Ryan U.M. (2011). Novel trypanosome *Trypanosoma gilletti* sp. (Euglenozoa: Trypanosomatidae) and the extension of the host range of *Trypanosoma copemani* to include the koala (*Phascolarctos cinereus*). Parasitology.

[21] Botero A., Cooper C., Thompson C.K., Clode P.L., Rose K., Thompson R.A. (2016). Morphological and phylogenetic description of *Trypanosoma noyesi* sp. nov.: An Australian wildlife trypanosome within the T. cruzi Clade. Protist.

[22] Ferreira J.I., da Costa A.P., Nunes P.H., Ramirez D., Fournier G.F., Saraiva D., Tonhosolo R., Marcili A. (2017). New Trypanosoma species, *Trypanosoma gennarii* sp. nov., from South American marsupial in Brazilian Cerrado. Acta Trop..

[23] Votýpka J., Stříbrná E., Modrý D., Bryja J., Bryjová A., Lukeš J. (2022). Unexpectedly high diversity of trypanosomes in small sub-Saharan mammals. Int. J. Parasitol..

[24] Marcili A., da Costa A.P., Soares H.S., Acosta Ida C., de Lima J.T.R., Minervino A.H.H., Melo A.T.L., Aguiar D.M., Pacheco R.C., Gennari S.M. (2013). Isolation and phylogenetic relationships of bat trypanosomes from different biomes in Mato Grosso, Brazil. J. Parasitol..

[25] Reynolds E.S. (1963). The use of lead citrate at high pH as an electron-opaque stain in electron microscopy. J. Cell Biol..

[26] Viola L.B., Campaner M., Takata C.S., Ferreira R.C., Rodrigues A.C., Freitas R.A., Duarte M.R., Grego K.F., Barrett T.V., Camargo E.P. (2008). Phylogeny of snake trypanosomes inferred by SSU rDNA sequences, their possible transmission by phlebotomines, and taxonomic appraisal by molecular, cross-infection and morphological analysis. Parasitology.

[27] Ferreira R.C., DE Souza A.A., Freitas R.A., Campaner M., Takata C.S.A., Barrett T.V., Shaw J.J., Teixeira M.M.G. (2008). A phylogenetic lineage of closely related trypanosomes (Trypanosomatidae, Kinetoplastida) of anurans and sand flies (Psychodidae, Diptera) sharing the same ecotopes in Brazilian Amazonia. J. Eukaryot. Microbiol..

[28] Lima L., Espinosa-Álvarez O., Hamilton P.B., Neves L., Takata C.S., Campaner M., Attias M., de Souza W., Camargo E.P., Teixeira M.M. (2013). *Trypanosoma livingstonei*: A new species from African bats supports the bat seeding hypothesis for the *Trypanosoma cruzi* clade. Parasites Vectors.

[29] Nicholas K.B.T., Nicholas H.B., Deerfield D.W. (1997). GeneDoc: Analysis and visualization of genetic variation. Embnew News..

[30] Stamatakis A. (2006). RAxML-VI-HPC: Maximum likelihood-based phylogenetic analyses with thousands of taxa and mixed models. Bioinformatics.

[31] Xia X., Xie Z., Salemi M., Chen L., Wang Y. (2003). An index of substitution saturation and its application. Mol. Phylogenet. Evol..

[32] Xia X., Lemey P., Lemey P., Salemi M., Vandamme A.-M. (2009). Assessing substitution saturation with DAMBE. The Phylo-Genetic Handbook: A Practical Approach to DNA and Protein Phylogeny.

[33] Xia X. (2013). DAMBE5: A comprehensive software package for data analysis in molecular biology and evolution. Mol. Biol. Evol..

[34] Stamatakis A. (2014). RAxML version 8: A tool for phylogenetic analysis and post-analysis of large phylogenies. Bioinformatics.

[35] Pattengale N.D., Alipour M., Bininda-Emonds O.R.P., Moret B.M.E., Stamatakis A. How many bootstrap replicates are necessary?. Proceedings of the 2009 IEEE International Conference on Bioinformatics and Biomedicine.

[36] Aberer A.J., Kobert K., Stamatakis A. (2014). ExaBayes: Massively parallel Bayesian tree inference for the whole-genome era. Mol. Biol. Evol..

[37] Rambaut A., Suchard M.A., Xie D., Drummond A.J. (2014). Tracer v1.6. http://beast.bio.ed.ac.uk/Tracer.

[38] Acosta I.D.C., da Costa A.P., Nunes P.H., Gondim M.F.N., Gatti A., Rossi J.L., Gennari S.M., Marcili A. (2013). Morphological and molecular characterization and phylogenetic relationships of a new species of trypanosome in *Tapirus terrestris* (lowland tapir), *Trypanosoma terrestris* sp. nov., from Atlantic Rainforest of southeastern Brazil. Parasites Vectors.

[39] Gruby M. (1843). Recherches sur une espèce observations nouvelle d’hématozoaire, Trypanosoma sanguinis. C. R. Acad. Sci..

[40] Paparini A., Macgregor J., Irwin P.J., Warren K., Ryan U.M. (2014). Novel genotypes of *Trypanosoma binneyi* from wild platypuses (*Ornithorhynchus anatinus*) and identification of a leech as a potential vector. Exp. Parasitol..

[41] Haag J., O’Huigin C., Overath P. (1998). The molecular phylogeny of trypanosomes: Evidence for an early divergence of the Salivaria. Mol. Biochem. Parasitol..

[42] Palma R.E., Jones M., Archer M., Dickman C. (2003). Evolution of American marsupials and their phylogenetic relationships with Australian metatherians. Predators with Pouches: The Biology of Carnivorous Marsupials.

[43] Ferreira R.C., Campaner M., Viola L.B., Takata C.S.A., Takeda G.F., Teixeira M.M.G. (2007). Morphological and molecular diversity and phylogenetic relationships among anuran trypanosomes from the Amazonia, Atlantic Forest and Pantanal biomes in Brazil. Parasitology.

[44] Lima L., da Silva F.M., Neves L., Attias M., Takata C.S., Campaner M., de Souza W., Hamilton P.B., Teixeira M.M. (2012). Evolutionary insights from bat trypanosomes: Morphological, developmental and phylogenetic evidence of a new species, *Trypanosoma (Schizotrypanum) erneyi* sp. nov., in African bats closely related to *Trypanosoma (Schizotrypanum) cruzi* and allied species. Protist.

[45] García L., Ortiz S., Osorio G., Torrico M.C., Torrico F., Solari A. (2012). Phylogenetic analysis of Bolivian bat trypanosomes of the subgenus *Schizotrypanum* based on cytochrome B sequence and minicircle analyses. PLoS ONE.

[46] Minter-Goedbloed E., Leake C.J., Minter D.M., McNamara J., Kimber C., Bastien P., Evans D.A., Le Ray D. (1993). Trypanosoma varani and T. grayi-like trypanosomes: Development in vivo and in insect hosts. Parasitol. Res..

[47] Cutolo A.A., Teodoro A.K.M., Ovallos F.G., Allegretti S.M., Galati E.A.B. (2014). Sandflies (Diptera: Psychodidae) associated with opossum nests at urban sites in southeastern Brazil: A risk factor for urban and periurban zoonotic Leishmania transmission?. Mem. Inst. Oswaldo Cruz.

[48] Quaresma P.F., Carvalho G.M.d.L., Ramos M.C.d.N.F., Filho J.D.A. (2012). Natural *Leishmania* sp. reservoirs and phlebotomine sandfly food source identification in Ibitipoca State Park, Minas Gerais, Brazil. Mem. Inst. Oswaldo Cruz.

[49] Deane M.P., Lenzi H.L., Jansen A. (1984). *Trypanosoma cruzi*: Vertebrate and invertebrate cycles in the same mammal host, the opossum *Didelphis marsupialis*. Mem. Inst. Oswaldo Cruz.

[50] Jansen A.M., Xavier S.C.d.C., Roque A.L.R. (2018). *Trypanosoma cruzi* transmission in the wild and its most important reservoir hosts in Brazil. Parasites Vectors.

[51] Silva E.O.d.R.e., Pattoli D.B.G., Camargo J. (1976). A new finding of *Trypanosoma (Megatrypanum) freitasi*, parasite of the opossum. Rev. Saude Publica.

[52] Lopes C.M.T., Menna-Barreto R.F.S., Pavan M.G., Pereira M.C.S., Roque A.L.R. (2018). *Trypanosoma janseni* sp. nov. (Trypanosomatida: Trypanosomatidae) isolated from *Didelphis aurita* (Mammalia: Didelphidae) in the Atlantic Rainforest of Rio de Janeiro, Brazil: Integrative taxonomy and phylogeography within the *Trypanosoma cruzi* clade. Memórias Inst. Oswaldo Cruz.

[53] Rodrigues M.S., Lima L., Xavier S.C.d.C., Herrera H.M., Rocha F.L., Roque A.L.R., Teixeira M.M.G., Jansen A.M. (2019). Uncovering *Trypanosoma* spp. diversity of wild mammals by the use of DNA from blood clots. Int. J. Parasitol. Parasites Wildl..

[54] Brandão E.M.V., Xavier S.C.C., Carvalhaes J.G., D’Andrea P.S., Lemos F.G., Azevedo F.C., Cássia-Pires R., Jansen A.M., Roque A.L.R. (2019). Trypanosomatids in small mammals of an agroecosystem in central Brazil: Another piece in the puzzle of parasite transmission in an anthropogenic landscape. Pathogens.

[55] Herrer A. (1942). *Trypanosoma phyllotis* sp. nov. e infecciones asociadas en uma titira, el *Phlebotomus nogushi*. Peru. Med. Exp. Salud Pública.

[56] Rodrigues V.L., Filho A.D.N.F. (1984). *Trypanosoma (Megatrypanum) rochasilvai*, sp. n., encontrada no Estado de São Paulo, Brasil, parasitando o *Oryzomys laticeps* (Leche, 1886) (Rodentia-Cricetidae). Rev. Bras. Biol..

[57] Herrera L., Urdaneta-Morales S. (1997). Synanthropic rodent reservoirs of *Trypanosoma cruzi* in the valley of Caracas, Venezuela. Rev. Inst. Med. Trop. Sao Paulo.

[58] Naiff R.D., Barrett T.V. (2013). *Trypanosoma* (*Megatrypanum*) *lainsoni* sp. nov. from *Mesomys hispidus* (Rodentia: Echimyidae) in Brazil: Trypomastigotes described from experimentally infected laboratory mice. Parasite.

[59] Pumhom P., Pognon D., Yangtara S., Thaprathorn N., Milocco C., Douangboupha B., Herder S., Chaval Y., Morand S., Jittapalapong S. (2014). Molecular prevalence of *Trypanosoma* spp. in wild rodents of Southeast Asia: Influence of human settlement habitat. Epidemiol. Infect..

[60] Hong X.-K., Zhang X., Fusco O.A., Lan Y.-G., Lun Z.-R., Lai D.-H. (2017). PCR-based identification of *Trypanosoma lewisi* and *Trypanosoma musculi* using maxicircle kinetoplast DNA. Acta Trop..

[61] Santos F.M., Barreto W.T.G., de Macedo G.C., Barros J.H.d.S., Xavier S.C.d.C., Garcia C.M., Mourão G., de Oliveira J., Rimoldi A.R., Porfírio G.E.d.O. (2019). The reservoir system for Trypanosoma (Kinetoplastida, Trypanosomatidae) species in large neotropical wetland. Acta Trop..

[62] Klink C.A., Machado R.B. (2005). A conservação do Cerrado brasileiro. Megadiversidade.

[63] Alho C.J.R., Mamede S.B., Benites M., Andrade B.S., Sepúlveda J.J.O. (2019). Threats to the biodiversity of the Brazilian Pantanal due to land use and occupation. Ambient. Soc..

[64] Roque A.L.R., D’andrea P.S., Jansen A.M., Duarte A.C.M., Xavier S.C.C., da Rocha M.G. (2008). *Trypanosoma cruzi* transmission cycle among wild and domestic mammals in three areas of orally transmitted Chagas disease outbreaks. Am. J. Trop. Med. Hyg..

[65] Keesing F., Belden L.K., Daszak P., Dobson A., Harvell C.D., Holt R.D., Hudson P., Jolles A.E., Jones K.E., Mitchell C.E. (2010). Impacts of biodiversity on the emergence and transmission of infectious diseases. Nature.

[66] Epstein P. (1997). The threatened plague. People Planet.

